# Severity as a moral qualifier of malady

**DOI:** 10.1186/s12910-023-00903-2

**Published:** 2023-03-31

**Authors:** Carl Tollef Solberg, Mathias Barra, Lars Sandman, Bjørn Hoffmann

**Affiliations:** 1 Centre for Medical Ethics (CME), Institute of Health and Society, Faculty of Medicine, University of Oslo, Kirkeveien 166, Postbox 1130, 0318 Oslo, Norway; 2 The Health Services Research Unit—HØKH, Akershus University Hospital HF, Lørenskog, Norway; 3 Department of Health, Medicine and Caring Sciences, The National Centre for Priorities in Health, Linköping University, Linköping, Sweden; 4 Institute for the Health Sciences, Norwegian University of Science and Technology, PO Box 191, N-2802 Gjøvik, Norway

**Keywords:** Decision making, Medical humanities, Philosophy, Rationing/resource allocation

## Abstract

The overarching aim of this article is to scrutinize how *severity* can work as a qualifier for the moral impetus of malady. While there is agreement that malady is of negative value*,* there is disagreement about precisely how this is so. Nevertheless, alleviating disease, injury, and associated suffering is almost universally considered good. Furthermore, the strength of a diseased person’s moral claims for our attention and efforts will inevitably vary. This article starts by reflecting on what kind of moral impetus malady incites. We then analyze how severity may qualify this impetus. We do so by discussing the relationship between severity and need, well-being and disvalue, death, urgency, rule of rescue, and distributive justice. We then summarize our thoughts about severity as a moral qualifier. We conclude that severity is, and should continue to be seen, as a morally significant concept that deserves continued attention in the future.

## Background

Malady^1^ is universally viewed as something bad; for individuals, groups, and society. Although there may be disagreement about what kinds of values are involved, there is agreement that malady is of negative value.^2^ Furthermore, instances of malady—say, a diseased person—seems to evoke, in most people, a moral impetus to alleviate disease and its consequences for others. Cure and care have motivated persons, professions, and institutions throughout recorded history [1]. Many ethical positions, such as consequentialism, deontology, virtue ethics, and proximity ethics, give rise to moral arguments for an obligation to intervene. It is also an incontrovertible fact that preventing and curing malady, as well as caring for the sufferer and alleviating malady, has been an integral feature of mystic rites, religious practices, all branches of philosophy, the natural sciences, and an omnipresent concern for individuals, professionals, institutions, and states [2].^3^

In the paragraph above, we have made two sweeping claims—that malady is universally a bad; and that curing or caring for the ill has permeated all human enterprise. Both claims can be challenged and can be qualified. It is easy to find accounts that ennoble the suffering, emphasize its capacity to build character, direct focus away from trivial matters, and allow its victims to emerge strengthened by escaping malady’s claw [3]. We claim that none of these suffering accounts contradicts the basic badness of malady—instead, such accounts point to a possible silver lining. Even if an individual is believed to *summa summarum* benefit from an encounter with malady, these accounts all recognize that there is something bad about malady, which nevertheless can be offset by other moral considerations. Malady is universally recognized as bad *in some sense or another*.

The second assertion should not be interpreted as that the alleviation of malady has been a concern for all instances of all human institutions. There will be counterexamples. Instead, this assertion merely points to the fact that, with very few exceptions, there are instances of concern for cure and care found within all levels of human interaction.

In this article, we assume that most cases of malady, considered in isolation, instill in fellow humans a *moral impetus* towards actions that mitigate the malady strickens’ unfortunate situation. This impetus’ strength, however, seems highly variable and will depend on several particulars.

One central factor, we argue, is how *severe* the malady is. The etymology of the term severity underscores its importance: The word itself can be traced back through the Latin ‘severus’ to the proto-Indo-European 'seg^h^,' meaning 'to hold' or to 'overpower.' The Latin ‘severus’ meant ‘extremely strict’, ‘extremely grievous,' or ‘exacting, painful.' In Norwegian, the corresponding term ‘alvorlig’ likely hails from Old Norse ‘all var’, loosely translatable as 'commanding your full being,' while the Swedish equivalent term ‘svår’ has its roots in Latin ‘serius,' which traces to ‘heavy,' or ‘grave.'^4^ Thus, something *severe* is serious, weighty, and adjures our undivided attention.

The concept of severity for priority setting purposes has also been subject to recent criticism by philosopher Daniel Hausman. Hausman follows several different lines of argument. He appears skeptical of a severity criterion for priority setting in health care because severity lacks moral justification and concludes that severity "is in need of moral justification" [4].

Several attempts to operationalize severity have been provided for priority setting purposes [5, 6]. Two notable examples are the *absolute QALY shortfall* (AQS) and the *proportional QALY shortfall* (PQS). We will not dig deep into the priority-setting debate here but use the opportunity to take one step back and discuss to what degree severity may or may not be of moral significance for action-guiding purposes.

The existence of multiple concepts of severity generates three partly conflicting hypotheses. Within moral philosophy, pluralism can be construed as evidence for either of two extreme positions: first, by following *the argument from relativity* by John Mackie [7], pluralism would suggest that the concept of severity itself is an error.^5^ At least, the fact that there exist variations of ‘severity’ may render it unsuited as a general moral qualifier of malady. Second, and by contrast, Derek Parfit’s metaphor of “climbing the same mountain on different sides” [8] suggests that people may approach the same severity construct from different directions. That is to say, the very same variations of severity that may instead indicate different approaches toward the same elusive concept. A third hypothesis is that there may be only specific (limited) conceptualizations of severity that can do the job as a moral qualifier of malady. The truth of the first hypothesis would weaken the impetus of severity, the second may strengthen it, and the third could provide a middle ground.

The aim of this article is to scrutinize how *severity* may qualify the moral impetus of malady. Our article proceeds as follows: after this introduction, we begin discussing what kind of moral impetus malady does incite. Next, we proceed to the main objective—an analysis of how severity may qualify the moral impetus of malady. We discuss the relationship between severity and need, well-being and disvalue, death, urgency, the rule of rescue, and distributive justice. We conclude that severity is, and should continue to be seen, as a morally significant concept that deserves continued attention.

## Discussion

### What kind of moral impetus does malady incite?

Being confronted with a person with a malady can potentially be action-guiding in different ways. While malady can be action-guiding for the person having the malady (not exposing others to infection, seeking help, adapting to an injury) [9], we focus on the impetus malady has on *others*. How do bystanders, family, or society act when someone is ill, and what is the moral component of these actions?

Furthermore, we believe that the moral content of actions guided by the moral impetus of malady is graded. A malady could generate a weak moral impetus towards acting, moderate in terms of a moral reason to help, strong in implying a duty, or very strong in terms of a moral imperative.^6^ Most European countries legally encode a general civil duty to help persons—often called a ‘rule to rescue’—when health or life itself is endangered. Their presence bears witness to deep-rooted cultural taboos against abandoning people in dire straits. Compared to general citizens, healthcare workers have a stronger legal obligation to aid.

Moreover, the moral impetus may be seen as supererogate or as fundamental and existential, for instance, by the phenomenology of intersubjective responsibility.^7^ Yet another way to justify a moral impetus of malady is from the perspective of justice, where malady interferes with a fair distribution of goods.^8^ In other words, that malady should be alleviated to secure fair opportunity or outcome in terms of a good and meaningful life in relation to other, more fortunate members of society.

The point we argue here is this: our shared culture indicates that another person’s malady provides moral reasons to act towards mitigating that someone’s condition. Confronted with malady, we often empathize with whoever has fallen ill; we wish them a speedy recovery or care and hope when recovery is impossible. For health professionals, diagnosing a person with a malady is action-guiding, where the actions are directed toward curing, reducing, or palliating the malady [9]. Depending on our proximity to the patient, we might also experience a moral obligation to ‘do something,’ even if acting requires a great sacrifice from us.^9^ [11].

While there may be disagreement on how strong the moral impetus of malady is, there seems to be unanimous agreement that malady carries *some* moral impetus for action. This moral impetus appears graded from weak to strong. Furthermore, it is first and foremost characterized by guiding us to act to alleviate the malady and the associated suffering, even when this comes at a (personal) cost.

### How does severity qualify the moral impetus of malady?

Not all maladies carry the same moral momentum. A common cold does not demand that we ‘drop everything and scramble to aid,' while most people will feel compelled to 'do something' faced with a life-threatening acute illness. Several notions exist about the magnitude of a malady’s impact on a person. This impact may be on their well-being, bodily function, life length, social standing, or role in their close or extended family. It is common to describe some maladies as severe (or serious).

In what follows, we identify different notions of severity related to the moral impetus of malady.^10^

#### Severity as a dimension of need

One central concept in healthcare is *need*. Many jurisdictions claim implicitly or explicitly to implement a needs-based healthcare system (in distinction to a demand-driven or something of sorts.) Need is an allusive concept, but a typical account of need builds on a ternary predicate *N(x,y,z)* to be interpreted as '*x* needs *y* for *z*.' In health care, then, a person *p* needs healthcare intervention *T* (treatment, care, etc.) to obtain the health benefit *B*—that is *N(p,T,B)—*if and only if there is a health-gap between *z*_*goa*l_ and *z*_*now*_ such that *T* would move *p* from *z*_*now*_ towards *z*_*goal*_ [12, 13]. In this framework, ‘severity’ can be understood in terms of the health gap z_now_ and _l_. Of course, *T* might not confer z_goal_ for p. There could be a difference between what p needs, and what *p* gets. Such a conceptualization requires a definition or delineation of health, an understanding of the healthcare system's goal in terms of health levels, and to what extent there is a gap in relation to the person's current situation. There is no severity when there is no gap, and hence no healthcare need either. On the other hand, this needs-account also includes the capacity to benefit, and following this, a person might have a condition with tremendous and incapacitating pain and suffering, but no healthcare intervention exists to reduce the pain and suffering. In such a situation, there is no moral impetus to act for the healthcare system in a needs-based healthcare system as there is no healthcare need (defined in terms of actionability). Hence, a needs-(and-actionability-) based conception of severity does not ascribe a moral impetus towards people in great pain and suffering if it cannot be addressed. On the other hand, the most severe condition can be addressed in some form, for instance, by providing comfort or by showing empathy.

#### Severity as lack of well-being and disvalue

There is a range of theories of well-being [14] that give different conceptions of severity. While (a) hedonistic theories of well-being define the severity of malady in terms of the magnitude of pain, (b) preference- and desire-based theories define severity in terms of frustrated preferences or desires, and (c) objective list theories will define severity in terms of *lack* of specific values, which are considered to be so independently of a person’s attitude towards them. Common to the different theories of well-being is that malady will induce negative well-being [15]. Hence, the stronger the negative well-being, the more severe the malady will be. The strength of the pain, frustration of preferences and/or desires, reduction in function, and number and characteristics of symptoms will all contribute to the severity of a malady.

Overall, a malady may give rise to two main forms of harm. One is *intrinsic* harm, that which is harmful in and of itself. For instance, pain is typically considered an intrinsic form of harm. Another critical form is *extrinsic* harm—not harmful in itself but rather because of its effects on other values. The latter type of harm is often formulated as *counterfactual (comparative) harm*: A malady can (factually) harm a person overall if that person (counterfactually) would have been better off had the malady not occurred.

#### Severity as death (losing one’s life)

If a person dies from a malady, this is often considered to be severe, and the relationship between severity and death thus merits separate discussion. By *death*, we do not mean the process of dying (which takes place within life) but rather the event of death (i.e., losing one's life.) It is uncontroversial to hold that the morbidity belongs to the diseased individual. Concerning the event of death itself, it is less clear who the victim is. Recall that it is common to hold that death is an irreversible event. Moreover, from a secular perspective, it is common to hold that death implies the permanent extinction of an individual (as there is no belief in an afterlife). In this sense, "being dead" cannot be intrinsically bad for an individual. Nevertheless, everyone agrees that a particular person’s death can be bad for the family, friends, and society left behind. This is not to say that all deaths inflict relatives adversely; sometimes, a slightly earlier death of a suffering relative can be perceived as a better (or the least bad) outcome. But the ambiguity surrounding if and how a person can be harmed by her own death,^11^ is not generally mirrored in ambiguity about if and how relatives of the deceased may be harmed.

Still, the orthodoxy within the so-called *badness of death* discourse in analytic philosophy is that death can be bad (and sometimes good) for those who die. Many hold that death is a particular type of counterfactual harm. The orthodoxy today is that death (i.e., the incident of death) cannot be intrinsically bad but only bad compared to the life you could have had, had you not died. This counterfactual account of the harm of death will generally imply that death is worse the earlier in life it occurs. That is to say, the counterfactual account of the harm of death takes a purely forward-looking perspective. This account captures the common intuition that since, in general, young individuals have more prospective good life left compared to older individuals, the death of the former will be worse than the death of the latter. In the context of severity, such an account of death implies that ceteris paribus, the death of young individuals, is more severe than the death of older ones. Moreover, the more likely a malady can result in death, the more severe it is. This again makes (the concept of) severity depend on prognosis, which can be uncertain or greatly variable amongst individuals in a group.

#### Severity as urgency

Etymologically, *urgency* relates to the Latin urge, which is related to "to press hard, push forward, force or drive." In the ethics literature, that which is urgent is pressing and demands quick action. As such, this seemingly overlaps with our etymological findings on severity. However, an’urgency’ has strong temporal connotations in everyday use. Something is urgent *now* and cannot remain urgent for very long. Concerning health care and malady, something is described as urgent when there is a narrow window to act either to (a) prevent further *irreversible* deterioration or (b) to salvage bodily function otherwise irretrievably lost. Severity and urgency, therefore, often coincide. From a secular perspective, death has an irreversible nature.^12^ Furthermore, even from religious perspectives, it is common to hold that death implies irreversible loss of earthly existence.^13^ Since irreversibility may be a necessary criterion (but not sufficient) for urgency. In fact, many cases of urgency will relate to death cases. There is no complete overlap between severity and urgency. A hypothermic finger may require urgent treatment to prevent the irreversible loss of the finger, but this does not automatically imply severity. In some circumstances, urgency and severity can even appear as independent notions. Cerebral infarction has become more urgent after the advent of modern anti-thrombolytic treatments [16] but arguably not more severe [17, 18]. As such, urgency appears more aligned with popular accounts of need than severity: something is urgent when you need it now. Other diseases, like ALS, are considered very severe but not urgent. Urgency, as such, seems to imply (i) irreversibility if no action is taken (ii) and an opportunity to mitigate. Both urgency and severity create an impetus toward action. While overlap may be seen (both in particular instances and concepts), urgency and severity also diverge. Hence, urgency cannot independently characterize severity as a moral qualifier for malady.

#### Severity and the rule of rescue

The “rule of rescue” has spawned its subliterature of bioethics [19]. Following Brock, “The Rule of Rescue (RoR) states if one can save people whose lives are imminently threatened, at reasonable cost or risk to oneself, one has a moral obligation to do so” [20]. Whether this so-called rule has any moral justification and it has spurred an ongoing discussion since it was first introduced into health policy ethics over 35 years ago [19, 21]. Objections to the RoR involve questioning whether it is a moral rule rather than a psychological trait unsupported by ethical argument. According to this view, the RoR causes us to unfairly favor identifiable patients over unidentified but equally needy patients [22]. There is general skepticism that a coherent version of the RoR can be formulated in health policy [23]. Notwithstanding, ethically and legally, the RoR applied to cases of malady is commonly viewed as a weak duty that only applies when an identifiable individual is in grave danger and when the prospective helper will not suffer unreasonable harm by providing aid [24, 25].

However, the rule of rescue is a powerful psychological motivation for individual behavior, sometimes called the ‘identifiable victim effect’ [26]. Moreover, it tends to involve severity somehow, rendering severity defined in terms of the rule of rescue circular. For example, The Australian Pharmaceutical Benefits Advisory Committee (PBAC)^14^ specifies one requirement for applying the Rule of Rescue to national pharmaceutical coverage decisions: "The medical condition defined by the requested restriction is severe, progressive, and expected to lead to premature death. The more severe the condition, or the younger the age at which a person with the condition might die, or the closer a person with the condition is to death, the more influential the rule of rescue might be in the consideration by PBAC.” [23, 28]. Hence, defining severity (and its moral impetus) in terms of the rule of rescue may lead to circularity.

#### Severity and distributive justice

Distributive justice has a comparative nature. The most frequent categories of distributional justice are utilitarianism, egalitarianism, prioritarianism, and sufficientarianism. Concerns for utilitarianism have already been discussed elsewhere in this article. Additionally, there have been further attempts to tie severity and distributive justice together. Accordingly, on egalitarianism, a negative value is put on inequality in and of itself, and the negative impact of malady to society cannot simply be added to the condition itself or its context but one must also account for how each person with a malady is contributing to the distribution of ill health. Thus, the egalitarian concern is relative in this sense. Moreover, prioritarianism emphazises aiding those absolutely worse off. In many cases, the malady may contribute to such worse off-ness. According to sufficientarianism, being below the sufficiency threshold might also add an extra moral impetus to severity.^15^

### Severity as a moral qualifier

In our coarse-grained analysis, we find that severity is a useful concept to describe the disvalue of malady, and we hypothesize that other ways to describe this disvalue can be translated into severity. This does not imply that severity fully corresponds to the moral impetus of malady since other factors will also plausibly impact whether the malady will be a call to action (e.g., resources.,^16^ efforts,^17^ size of benefit,^18^ relational factors, factors related to desert, etc.).

Our analysis has not allowed us to put a number on the moral impetus of severity. Such an endeavor would necessarily include severity's definition, possibly a method for severity's measurement (if severity comes in degrees), and an ethical argument to support why severity should invoke such a moral impetus. In specific areas, e.g., for particular diseases, severity indexes have been developed and applied. Instead, we have explored how various ethical arguments can defend different accounts for why and how severity may be a moral qualifier for this impetus.

In being a central factor for describing the disvalue of malady, and thereby central for the moral impetus—severity will have an important role in different settings where malady should result in a call for action—be it in the clinical situation, as a bystander to someone taken ill outside the healthcare system or for priority setting of resource to and within healthcare.

## Conclusions

The aim of this study was to scrutinize how severity may qualify the moral impetus of malady.

**Fig. 1:**
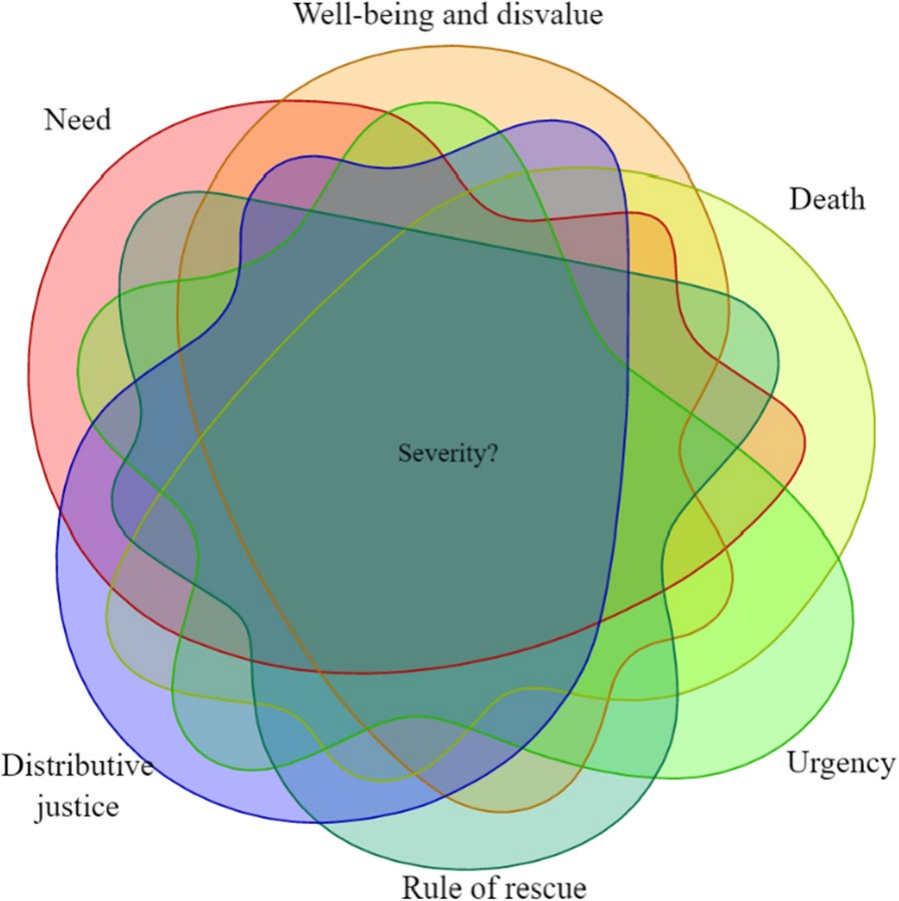
A Venn-diagram to illustrate the overlap between the various conceptions of severity with moral relevance

We have looked at the relationship between severity on the one hand and a set of six selected phenomena that are commonly considered morally significant on the other hand (i.e., need, well-being and disvalue, death, urgency, rule of rescue, and distributive justice.) We probed the "mountain from different angles" hypothesis by approaching severity and each of the respective morally significant phenomena through a Venn diagram line of thinking, see Fig. 1. By searching for overlap between severity and the six morally relevant concepts, we have moved closer to a conceptual core of severity: a shared aspect of all six phenomena seems to be that they are associated with accounts of severity *and* impart a moral impetus for action. Based on our analysis, we conclude that severity likely is a morally significant phenomenon. Severity as a moral qualifier will have implications for health policy, priority setting, and the professional-patient relationship, as it directs the moral impetus towards individual patients, groups of patients, and persons, as well as the health of populations. Nevertheless, we remain agnostic on the moral significance of severity for specific priority-setting purposes, and we are open to the shortcomings of the current priority-setting operationalizations of severity. That being said, we conclude that severity is a morally significant concept that deserves continued attention.

## Data Availability

Not applicable.

## Abbreviations

AQS: Absolute QALY shortfall
PBAC: The Australian Pharmaceutical Benefits Advisory Committee
PQS: Proportional QALY shortfall
QALY: Quality-adjusted life year

## Footnotes

1 We use *malady* as an umbrella for a wide range of terms, such as "disease," "injury," "illness," “sickness," and other related terms (1).

2 That is to say; people perceive maladies as of negative value, which again presumes that there are reasons to seek to alleviate maladies.

3 This concern is there, perhaps, because the malady itself is a ubiquitous fact of life.

4 See “seǵ," "severus” or “serius” in Wiktionary, or “severe” in Oxford Dictionary of English Etymology.

5 The argument from relativity is often referred to as *the argument from disagreement*.

6 In moral philosophy, one has been more concerned with moral dilemmas and seemingly insolvable challenges at the margin of life than with everyday moral practices where consensus is implicit.

7 E.g., in the philosophy of Emanuel Levinas, where the face of another person (the Other) constitutes a moral claim-making ethics. Here, ethics is the first philosophy.

8 E.g., A. Caplan: "disease and disability become the object of concern in Western society because they are seen as a threat to equal opportunity, and in turn to the moral foundation of economic life" (Caplan [10], p. 236).

9 Live kidney donors are, for example, mainly parents, spouses, or siblings (Ralph et al. 2019) .

10 One reviewer suggested that we categorize severity in line with "subjective" and "objective." We agree that the subjective–objective dichotomy is a valuable distinction in philosophy for many purposes. However, in the context of this article, there is a risk that the distinction would instead make our discussion of dimensions of severity more rigid rather than add a novel perspective to our inquiry. What is more, if we were to use these two concepts, we would have to clearly define "objective" (e.g., mind-independent) and stick with that definition throughout the article.

11 With "death," we here mean the "incident of death" rather than "the process of dying." Of course, the process of dying (which only takes place while still alive) can be bad for those who die.

12 That is to say, from a secular perspective, it is common to presume *the termination thesis*—which states that death implies the irreversible loss of existence.

13 One notable exception is death’s irreversibility is, of course, the belief in reincarnation. Another more secular exception is the belief in cryonics.

14 This is one of few explicit references to the RoR. However, it has been observed that other jurisdictions (e.g., England and its HTA-authority NICE) might use it implicitly. See [27].

15 An additional use of severity enables a breach of otherwise accepted norms: e.g., allowing late-term termination of pregnancy when a severe fetal condition is diagnosed.

16 Resources, broadly construed, are one crucial factor for the strength of the moral impetus.

17 When the efforts required to alleviate malady are sufficiently low, we believe that most people would experience a moral impetus to act to mitigate malady.

18 Another factor of this impetus is the size, in some sense, of the benefit the intervention would impart (recall the above discussion on need).

## References

[1] Clouser KD, Culver CM, Gert B (1981). Malady: a new treatment of disease. Hastings Cent Rep.

[2] Porter D. Health, civilization and the state: a history of public health from ancient to modern times. [Internet]. 2005 [cited 2022 Apr 21]. Available from: http://public.ebookcentral.proquest.com/choice/publicfullrecord.aspx?p=241913. 10.1136/bmj.318.7190.1083 PMC1115477 10205130

[3] Fitzpatrick SJ, Kerridge IH, Jordens CFC, Zoloth L, Tollefsen C, Tsomo KL (2016). Religious perspectives on human suffering: implications for medicine and bioethics. J Relig Health.

[4] Hausman D (2019). The significance of ‘severity’. J Med Ethics.

[5] Shah KK (2009). Severity of illness and priority setting in healthcare: a review of the literature. Health Policy.

[6] Barra M, Broqvist M, Gustavsson E, Henriksson M, Juth N, Sandman L (2020). Severity as a priority setting criterion: setting a challenging research agenda. Health Care Anal.

[7] Mackie JL (1990). Ethics: inventing right and wrong.

[8] Parfit D (2011). On what matters.

[9] Hofmann B. Acknowledging and addressing the many ethical aspects of disease. Patient Educ Couns. 2021; Available from: https://www.sciencedirect.com/science/article/abs/pii/S0738399121006248. 10.1016/j.pec.2021.09.015 34625319

[10] Caplan AL (1993). The concepts of health, illness, and disease. Companion encyclopedia of the history of medicine.

[11] Fellner CH, Marshall JR (1970). Kidney donors—the myth of informed consent. Am J Psychiatry.

[12] Gustavsson E. Characterising Needs in Health Care Priority Setting [Internet] [Ph.D.]. [Linköping, Sweden]: Linköping University; 2018. Available from: http://urn.kb.se/resolve?urn=urn:nbn:se:liu:diva-144207.

[13] Gustavsson E (2019). Patients with multiple needs for healthcare and priority to the worse off. Bioethics.

[14] Oades LG, Mossman L (2017). The science of wellbeing and positive psychology. Wellbeing recovery and mental health.

[15] Crisp R. Well-being. Standford Encycl Philos 2021;2021. Available from: https://plato.stanford.edu/cgi-bin/encyclopedia/archinfo.cgi?entry=well-being.

[16] Gomez CR (1993). Editorial: Time is brain!. J Stroke Cerebrovasc Dis..

[17] Carandang R, Seshadri S, Beiser A, Kelly-Hayes M, Kase CS, Kannel WB (2006). Trends in incidence, lifetime risk, severity, and 30-day mortality of stroke over the past 50 years. JAMA..

[18] Labberton AS, Rønning OM, Thommessen B, Barra M (2019). Changes in survival and characteristics among older stroke unit patients-1994 versus 2012. Brain Behav..

[19] McKie J, Richardson J (2003). The rule of rescue. Soc Sci Med..

[20] Brock DW (2015). Identified versus statistical lives: some introductory issues and arguments. Identified versus statistical lives: an interdisciplinary perspective.

[21] Jonsen AR (1986). Bentham in a box: technology assessment and health care allocation. Law Med Health Care.

[22] Lübbe W (2019). Appeal to the Rule of Rescue in health care: discriminating and not benevolent?. Med Health Care Philos..

[23] Cookson R, McCabe C, Tsuchiya A (2008). Public healthcare resource allocation and the Rule of Rescue. J Med Ethics.

[24] Weinrib EJ (1980). The case for a duty to rescue. Yale Law J.

[25] Rulli T, Millum J (2016). Rescuing the duty to rescue. J Med Ethics.

[26] Jenni KE, Loewenstein G (1997). Explaining the “Identifiable Victim Effect”. J Risk Uncertain..

[27] Charlton V (2022). Does NICE apply the rule of rescue in its approach to highly specialised technologies ?. J Med Ethics..

[28] Rawlins MD, Culyer AJ (2004). National Institute for Clinical Excellence and its value judgments. BMJ.

